# A Dose-Dependent Study Examining Dexmedetomidine’s Possible Effects Against Oxidative, Fibrotic, and Apoptotic Damage Induced by Radiation Exposure in Spleen Tissue

**DOI:** 10.3390/life15091430

**Published:** 2025-09-12

**Authors:** Hatice Beyazal Polat, Hamit Yılmaz, Kagan Kilinc, Belemir Gülhan, Sema Yılmaz Rakıcı, Levent Tümkaya

**Affiliations:** 1 Department of Internal Medicine, Faculty of Medicine, Recep Tayyip Erdogan University, Rize 55139, Türkiye; 2 Department of Biophysics, Faculty of Medicine, Kahramanmaraş Sütçü İmam University, Rize 46050, Türkiye; hamit.yilmaz@erdogan.edu.tr; 3 Department of Genetic and Bioengineering, Faculty of Engineering and Natural Sciences, Gumushane University, Gumushane 29000, Türkiye; 4 Department of Histology and Embryology, Faculty of Medicine, Samsun University, Samsun 55000, Türkiye; 5 Radiation Oncology, Recep Tayyip Erdogan University, Rize 53100, Türkiye; sema.rakici@erdogan.edu.tr; 6 Department of Histology and Embryology, Faculty of Medicine, Ondokuz Mayıs University, Samsun 55139, Türkiye; levent.tumkaya@omu.edu.tr

**Keywords:** radiotherapy, dexmedetomidine, apoptosis, fibrosis, white pulp, red pulp

## Abstract

**Objective:** This study aimed to investigate the potential splenic tissue damage induced by radiotherapy (RT) and the potential protective effect of different doses of dexmedetomidine on this damage at the histopathological, immunohistochemical, and biochemical levels. **Materials and Methods:** In our study, Sprague Dawley rats were randomly divided into four groups: Control, Radiotherapy (RT; 8 Gy), RT + Dexmedetomidine 100 µg/kg (RT-D100), and RT + Dexmedetomidine 200 µg/kg (RT-D200). A single dose of 8 Gy radiotherapy was administered to each RT group. Spleen tissues were examined histologically with hematoxylin-eosin and immunohistochemically with anti-Caspase-3, anti-TGF-β1, and anti-TGF-β3 using light microscopy. TBARS and total thiol levels were also analyzed to assess oxidative stress and antioxidant capacity. **Results:** Histopathological results showed a significant decrease in white pulp diameter, decreased cellular density, and increased congestion in the red pulp in the RT group. Significant fibrosis, sinusoidal dilatation, vacuolization, and amyloid deposition were detected in the white pulp in the RT group. Regarding anti-caspase-3 immunoreactivity, strong positivity increased in the red pulp in the RT group, while a significant increase was observed in the white pulp in both the RT-D100 and RT groups. While the proportion of TGF-β1 immunopositive cells did not change significantly in the RT group, they increased significantly in both dexmedetomidine groups (especially RT-D200). TGF-β3 expression increased significantly only in the RT-D100 group. In biochemical analyses, TBARS levels increased significantly in the RT-D100 group. Total thiol levels decreased in the RT group and increased in the dexmedetomidine-treated groups. **Conclusions:** While RT caused histopathological damage and increased oxidative stress in spleen tissue, dexmedetomidine reduced this damage in a dose-dependent manner. The different immunohistochemical profiles of TGF-β1 and TGF-β3 suggest that these cytokines may have different functions in the spleen. 100 µg/kg dexmedetomidine stimulates a regenerative response through TGF-β3, while 200 µg/kg dexmedetomidine may provide immune regulation and antioxidative defense through TGF-β1.

## 1. Introduction

Radiotherapy, an effective treatment method for many malignancies, continues throughout the cancer treatment process [1]. It has been reported that radiotherapy causes oxidative stress by inducing reactive oxygen radicals (ROS) at the cellular level [2]. The direct effect of ionizing radiation on the human body is cytological and manifests as cell death. Cell death resulting from ionizing radiation occurs in interphase and proliferative death. Interphase refers to cells that stop dividing after radiation and begin to die. High doses of radiation cause chromatin degradation. In proliferative death, cells forfeit their capacity to proliferate following multiple division cycles and subsequently commence apoptosis [3]. The cytotoxic effects of radiation occur through direct and indirect effects on DNA molecules. In direct radiation, radiation strikes the DNA molecule, causing structural damage, while in indirect radiation, it strikes water molecules within the cell, releasing free radicals. Free radicals, characterized by the presence of an unpaired electron, cause damage to the DNA molecule [4]. The resulting oxidative stress leads to disruption of the cellular redox balance and inadequacy of endogenous defense systems [5]. Additionally, ROS serves a dual function in cell physiology. Excessive accumulation of ROS can trigger oxidative stress, playing a role in the pathophysiology of many diseases. On the other hand, they are involved in redox homeostasis, immune responses, or DNA repair [6].

During the apoptosis process, the levels of antioxidant enzymes decrease, while the amount of lipid peroxidation products (such as malondialdehyde) increases [7]. Additionally, radiation triggers apoptosis in the spleen, leading to DNA damage and iron accumulation [8,9,10]. The hematopoietic system is highly susceptible to radiation damage. Mature white blood cells and hematopoietic progenitor cells undergo apoptosis due to high doses of radiation. Reticulocytes and red blood cells lack DNA and apoptotic machinery and therefore undergo hemolysis, which occurs due to the oxidation and denaturation of hemoglobin [10]. However, low-dose radiation not only triggers cellular repair mechanisms in the spleen but also sometimes induces apoptosis even at very low doses, leading to disorders in immune system cells [9,11]. Radiotherapy at a dose of 8 Gy has been reported to cause late hematopoietic effects and splenomegaly within 12–16 months [12].

The Transforming Growth Factor Beta (TGF-β) family, particularly TGF-β1 and TGF-β3, plays an important role in tissue responses after radiation. Following radiation, TGF-β1 and TGF-β3 levels increase, triggering fibrosis, inflammation, and tissue repair processes [13,14,15]. TGF-β1, which has immunosuppressive effects, plays a role in regulating the post-treatment immune response in the tumor microenvironment [16,17]. The effects of radiotherapy include the role of the TGF-β family in tissue repair and immune modulation, as well as the induction of cellular death processes through Caspase-3 [18,19].

One of the effective agents in preventing inflammation and oxidative damage caused by radiotherapy is dexmedetomidine [20,21]. Dexmedetomidine, which specifically targets alpha-2 adrenergic receptors, reduces sympathetic activity, leading to an analgesic effect [22]. Dexmedetomidine modulates the expression of immune markers in the spleen and reduces the secretion of inflammatory cytokines [23]. Dexmedetomidine was chosen in our study because it has anti-inflammatory effects and is an effective option for inflammatory diseases [23]. In this context, our study aims to investigate the effects of 100 µg/kg and 200 µg/kg dexmedetomidine on spleen tissue exposed to 8 Gy radiotherapy and the possible underlying mechanisms using histopathological, immunohistochemical, and biochemical methods.

## 2. Materials and Methods

### 2.1. Ethical Approval and Animals

The current experimental study was conducted with approval from the Recep Tayyip Erdoğan University Animal Experiments Ethics Committee (Rize, Turkey) dated 16 April 2018, numbered 2018/31, and spleen tissues were used in this study. In this experimental study, the experimental procedures were completed in accordance with the ARRIVE (Animal Research: Reporting of In Vivo Experiments) guideline, which aims to increase transparency and accuracy in the reporting of animal experiments. Rats were obtained from the Recep Tayyip Erdoğan University Animal Experiments Application Unit. All rats were kept under hygienic conditions at 55–60% humidity and room temperature (mean 22 °C) and had access to tap water and pellet chow ad libitum. Thirty-two 5-6-month-old male Sprague Dawley rats (340 ± 60 g) were used in the study. The number of rats was determined according to the results of power analysis. Rats were randomly divided into four groups (*n* = 8): Control group (Cont), Radiotherapy group (RT), 100 µg/kg dexmedetomidine (Precedex, Hospira, Lake Forest, IL, USA) administered before RT (RT-D100), 200 µg/kg dexmedetomidine (Precedex, Hospira, Lake Forest, IL, USA) administered before RT (RT-D200). Dexmedetomidine was administered at doses of 100 and 200 µg/kg (i.p.). These doses were chosen based on the radioprotective, antioxidant, and anti-inflammatory effects reported. Dexmedetomidine at 100–200 µg/kg has been shown to reduce tissue oxidative damage and functional impairment in ionizing radiation models; therefore, in our spleen-specific study, we selected 100 µg/kg as the effective dose and 200 µg/kg, a higher dose, to test a similar dose–response window. In our study, no clinically significant adverse events were detected under systematic follow-up. Rats in the RT groups were exposed to 8 Gy single-fraction whole-body irradiation. 100 µg/kg Dexmedetomidine (dissolved in 1 mL of physiological saline solution) was injected i.p. into the RT-D100 group, and 200 µg/kg Dexmedetomidine (dissolved in 1 mL of physiological saline solution) was injected i.p. into the RT-D200 group 30 min before radiotherapy exposure. The control group received 1 mL of physiological saline solution i.p. Following the experimental procedures, rat tissues were dissected under deep anesthesia.

### 2.2. Radiotherapy Procedure

Ketamine hydrochloride (Ketalar^®^, 50 mg/kg, Pfizer İlaçları Ltd. Şti, Istanbul, Turkey) and xylazine hydrochloride (Rompun^®^, 5 mg/kg, Bayer, USA) were used for anesthesia. All subjects were anesthetized with 50 mg/kg ketamine HCl and 10 mg/kg xylazine HCl 24 h after the X-ray procedure and sacrificed by anesthesia overdose for the collection of the specimens needed for the experiment. Following planning CT scans, conformal planning was performed on anesthetized rats using the CMS Xio (v5.0, Elekta, Stockholm, Sweden) system. External beam radiotherapy was administered at a dose of 8 Gy using a linear accelerator (Elekta Synergy) at 100 cm skin source distance (SSD), accompanied by a 0.5 cm bolus and 6 MV photon energy [19]. A single fraction of 8 Gy was administered using a linear accelerator (6 MV X-rays). The rats were positioned supine and administered in an anterior–posterior-posterior–anterior configuration, ensuring a homogeneous dose distribution throughout the body. Rats were euthanized by decapitation under deep anesthesia, in accordance with ethical guidelines for the care and use of laboratory animals.

### 2.3. Tissue Processing and Histopathological Evaluation

Half of the spleen tissue removed from each rat was fixed for immunohistochemical and histological processing. The other half was used for biochemical analyses. The dissected spleen tissues were incubated in 10% formalin solution for 10 days before the fixation process was completed. After being washed overnight in running water, the tissues were dehydrated by rinsing them with graded alcohols (70%, 80%, 96%, and 100%). The tissues were then cleared with xylene and incubated in liquid paraffin at 60 °C to complete the infiltration. Following routine tissue processing, 5-µm-thick sections were obtained from paraffin-embedded sections using a microtome and stained with hematoxylin and eosin. Sections were evaluated using the CellSens Entry software, version 1.18 (Olympus Corporation, Tokyo, Japan) on a light microscope equipped with a camera (Olympus, BX43, Center Valley, PA, USA). White pulp diameter was measured using the planimetric method in the histopathological images obtained from each section (cellSens Entry microscope software program, Olympus, Center Valley, PA, USA). The diameter of the follicles in each image was measured, and the average was calculated. Histopathological scoring was based on a category showing an ordered progression: 0—absent, 1—mild, 2—moderate, 3—severe.

### 2.4. Immunohistochemical Analyses

4 µm-thick sections from paraffin blocks were stained with primary antibodies against anti-TGF-β1 (ab66043, Abcam, Cambridge, UK, dilution: 1/100), anti-TGF-β3 (ab15537, Abcam, Cambridge, UK, dilution: 1/100), and anti-Caspase 3 (ab4051, Abcam, USA, dilution: 1/300). A mouse-specific HRP-AEC kit (ab93705, Abcam, Cambridge, UK) was used for staining. Mayer’s hematoxylin was used for counterstaining. A camera-mounted microscope (Olympus, BX43, Center Valley, PA, USA) was used to obtain images of the sections. A semi-quantitative scoring method was used to evaluate immunoreactivity in all groups: Reactions were graded as “mild,” “modified,” or “strong” based on staining intensity. Positive reactions were scored as “%.” [22]. The evaluation was performed blindly by an expert histologist. ImageJ software (version 1.54; NIH, Bethesda, MD, USA) was used on microscopic images to perform immunohistochemical analyses.

### 2.5. Biochemical Tissue Sampling and Homogenization

For biochemical analyses, spleen tissues were removed, washed with saline solution, and stored at −80 °C. A mixture of 1 L of 20 mM sodium phosphate and 140 mM potassium chloride was prepared for tissue homogenization (pH 7.4). After 100 mg of tissue sample was added to 1 mL of the resulting homogenization solution, it was centrifuged at 800× *g* for 10 min at 4 °C. The resulting supernatant was used in the determination of Thiobarbituric acid reactive substances (TBARS) and total thiol. Spectrophotometric evaluation was performed. Spectrophotometric measurements were performed using the Thermo Scientific Multiskan GO instrument (Vantaa, Finland). TBARS determination was performed according to the modified method of Ohkawa et al. The principle of the method is based on the formation of a pink-colored complex upon heating of malondialdehyde (MDA), a product of lipid peroxidation, with thiobarbituric acid (TBA) solution [24]. 200 µL of standards and supernatants were pipetted, and 50 µL of 8.1% SDS (Sigma-Aldrich, St. Louis, MO, USA), 375 µL of 20% acetic acid (pH = 3.5) (MERCK), and 375 µL of 0.8% TBA (MERCK) were added. The mixture was vortexed and then incubated in a boiling water bath for 1 h. After incubation, it was cooled in ice water for 5 min and centrifuged at 750 g for 10 min. The resulting pink color was read at 532 nm on a Thermo Scientific Multiskan GO instrument (Vantaa, Finland), a standard graph was plotted, and concentrations were calculated. Results were calculated as nmol/g tissue. Determination of Total Thiol (TT) Groups: The determination of total thiol groups is based on the spectrophotometric measurement of the yellow color produced by free sulfhydryl groups using Ellman’s reagent [25,26]. 100 µL of 3 M Na_2_HPO_4_ (HIMEDIA) and 25 µL of DTNB (Sigma-Aldrich) were added to 25 µL pipetted standards and supernatants. Following gentle shaking, the resulting yellow color was read at 412 nm on a Thermo Scientific Multiskan GO instrument (Vantaa, Finland), a standard graph was plotted, and concentrations were calculated. Results were calculated as mM/g tissue.

### 2.6. Statistical Analyses

Statistical analyses of the data were performed using GraphPad Prism (version 10.2, GraphPad Software, San Diego, CA, USA). Numerical data were expressed as mean ± standard deviation (mean ± SD). The Shapiro–Wilk test was used to assess whether the data were normally distributed. One-way analysis of variance (One-way ANOVA) was used to determine the differences between normally distributed groups, and Tukey’s post hoc test was used to determine which groups had significant differences. For data that did not show normal distribution, comparisons between groups were made using the Kruskal–Wallis test. A *p*-value of <0.05 was considered statistically significant.

## 3. Results

### 3.1. Histopathological Findings

**Figure 1:**
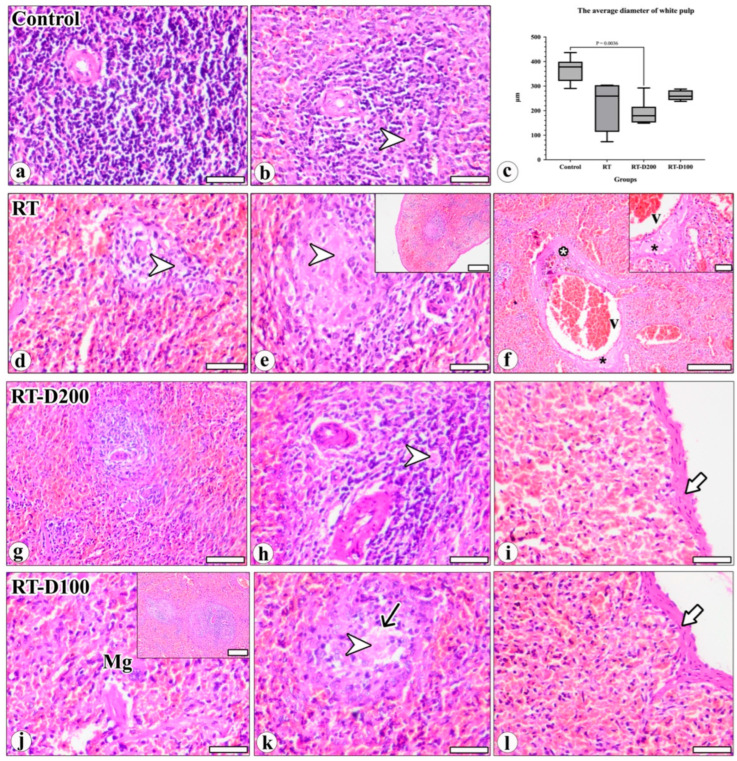
(**a**–**l**) Light microscopic images obtained from hematoxylin-eosin-stained sections of all groups are shown. (**c**) The mean diameter of the white pulp of all groups is shown. A decrease in white pulp diameter (µm) is observed in the RT-D100 group compared to the Control group (*p* = 0.0036). (**b**,**d**,**e**,**h**,**k**) Fibrosis areas (arrowhead) are observed in the groups. (**f**) In the RT-D200 group, disruption of the connective tissue around the vessels and wall thickening (white asterisk) are noticed. The upper left inset shows stromal degeneration and vacuolization (black asterisk) in the tissue surrounding the vessel. This can be selected as a histopathological marker of inflammation. In addition, the homogeneous, amorphous, pale pink appearance seen in the interstitial space around the vessels is thought to be due to amyloid deposition. (**k**) The thin arrow shows the decrease in cell density due to vacuolization seen in the white pulp. (**i**,**l**) The white arrow indicates the spleen capsule. It is noteworthy that the capsule structure is preserved. Mg: Megakaryocyte, white arrow: spleen capsule, V: Vessel. Scale bars: (**a**,**b**,**d**,**e**,**g**–**l**) 40 µm, (**e**,**j**) (image in the upper right corner): 200 µm, (**f**) 200 µm, (**f**) (image in the right corner): 40 µm. All parameters were semi-quantitatively scored on a scale from 0 to 3 (0 = absent, 1 = mild, 2 = moderate, 3 = severe).

In sections stained with hematoxylin-eosin, the spleen in the control group maintained its normal histological organization, and the white pulp areas were prominent and rounded (Figure 1a,b). In the radiotherapy (RT) group, a significant decrease in white pulp diameter, decreased cellular density, and congestion in the red pulp areas were observed (Figure 1d–f). Relative improvements in tissue morphology were observed in the RT-D100 and RT-D200 groups due to dexmedetomidine administration (Figure 1g–l). It was particularly notable that the white pulp diameter was more prominent in the RT-D100 group, and congestion decreased (Figure 1j–l). According to morphometric analysis results, the white pulp diameter decreased significantly in the RT group (Figure 1d–f), and this constriction was partially resolved in the RT-D100 group (*p* < 0.0001) (Figure 1j–l).

**Figure 2:**
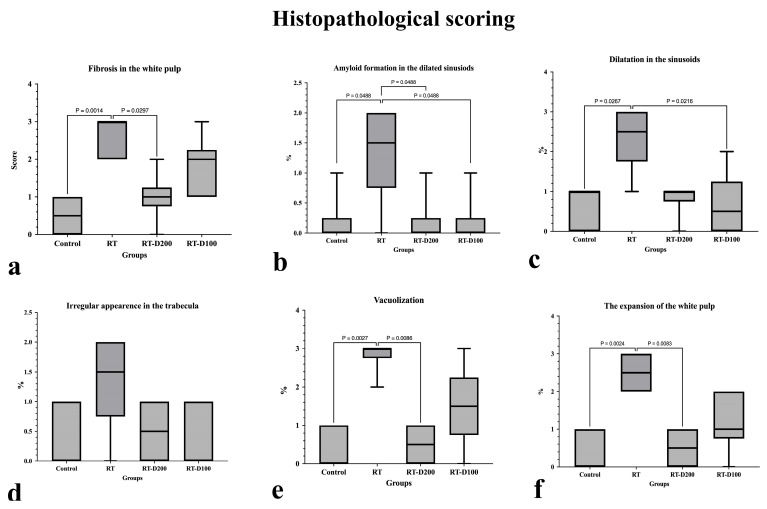
(**a**–**f**) Graphs containing histopathological evaluation of all groups. (**a**–**c**,**e**,**f**) Differences between groups at *p* < 0.05 and *p* < 0.01 levels are shown in the graph.

**Figure 3:**
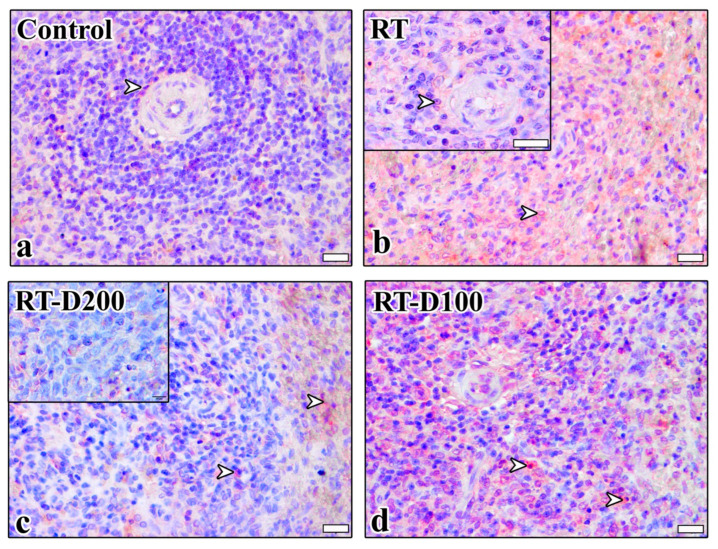
(**a**–**d**) Anti-caspase 3 immunoreactions are seen in all groups. Scale bars: 20 µm. Positive staining is indicated by arrowheads. Mayer’s hematoxylin was used for counterstaining. A semi-quantitative scoring system was used for evaluation: <10% = negative, 10–29% = mild, 30–59% = moderate, 60–100% = severe immunopositive cells.

According to histopathological scoring results, significant morphological changes were observed in the spleen tissue in the radiotherapy (RT) group compared to the control group. The fibrosis score in the white pulp increased significantly in the RT group (*p* = 0.0014). A significant decrease was observed in the RT-D200 groups compared to the RT group (*p* = 0.0297). Amyloid formation in dilated sinusoids was significantly increased in the RT group, and this increase was significant compared to both the control and treatment groups (*p* = 0.0488). Sinusoid dilatation increased significantly in the RT group compared to the control and RT-D100 groups (respectively, *p* = 0.0267; *p* = 0.0216). No significant difference was observed between groups in terms of trabecular irregularity. Vacuolization scores increased significantly in the RT group compared to the control group (*p* = 0.0027). This decreased significantly in the RT-D200 group, with a statistically significant difference compared to the RT group (*p* = 0.0086). A significant increase in white pulp expansion was observed in the RT group compared to the control group (*p* = 0.0024). This increase was significantly reduced in the RT-D200 group (*p* = 0.0083) (Figure 2a–f). It is observed that the anti-caspase-3 reaction is at the nuclear level in the Control and RT groups, while it is stained intensely at the nuclear level and occasionally at the cytoplasmic level in the RT-D100 and RT-D200 groups (Figure 3).

### 3.2. Immunohistochemical Findings

**Figure 4:**
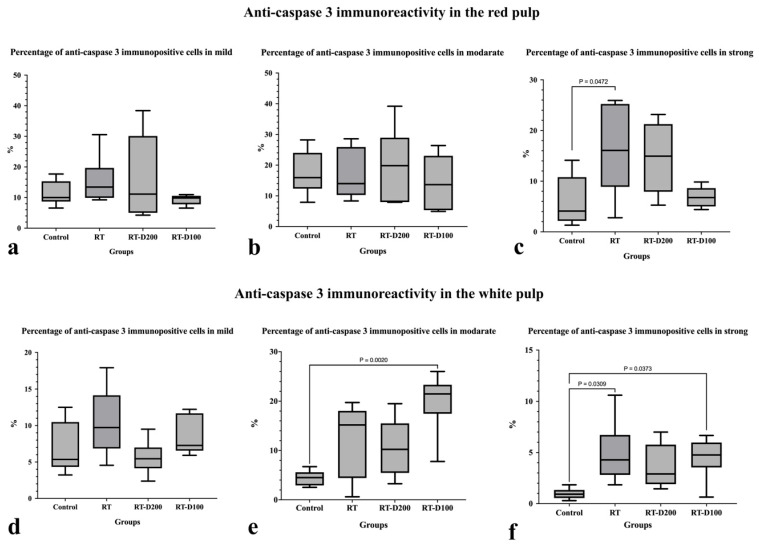
(**a**–**f**) Graphs containing anti-caspase 3 immunoreactivity assessment of all groups. (**c**,**e**,**f**) Differences between groups at the *p* < 0.05 level are shown in the graph.

When the percentage of mildly positive cells for anti-caspase-3 immunoreactivity in the red pulp was evaluated, no significant difference was observed between groups (Figure 3 and Figure 4a). Similarly, no significant difference was found between the groups in the percentage of moderately positive lymphocytes and macrophages (Figure 3 and Figure 4b). However, a significant increase in the strong positivity level was observed in the RT group compared to the control group (*p* = 0.0472) (Figure 3 and Figure 4c). When anti-caspase-3 immunoreactivity in the white pulp was examined, no significant difference was observed in the percentage of mildly lymphocytes and macrophages (Figure 3 and Figure 4d). The percentage of moderately lymphocytes and macrophages was significantly increased in the RT-D100 group compared to the control group (*p* = 0.002) (Figure 3 and Figure 4e). When evaluated in terms of strong positivity, a significant increase was observed in the RT and RT-D100 groups compared to the control group (respectively, *p* = 0.0309 and *p* = 0.0373) (Figure 3 and Figure 4f).

**Figure 5:**
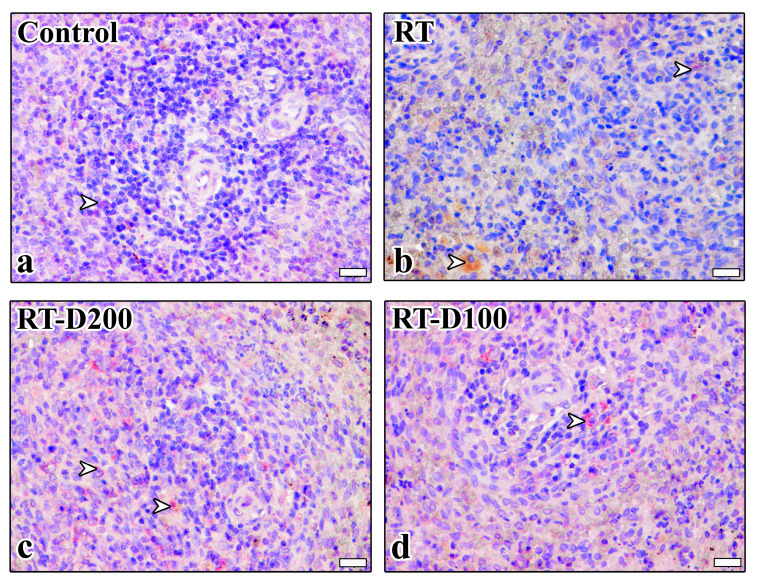
(**a**–**d**) Anti-TGF β1 immunoreactions are seen in all groups. Scale bars: 20 µm. Positive staining is indicated by arrowheads. Mayer’s hematoxylin was used for counterstaining. A semi-quantitative scoring system was used for evaluation: <10% = negative, 10–29% = mild, 30–59% = moderate, 60–100% = severe immunopositive cells.

**Figure 6:**
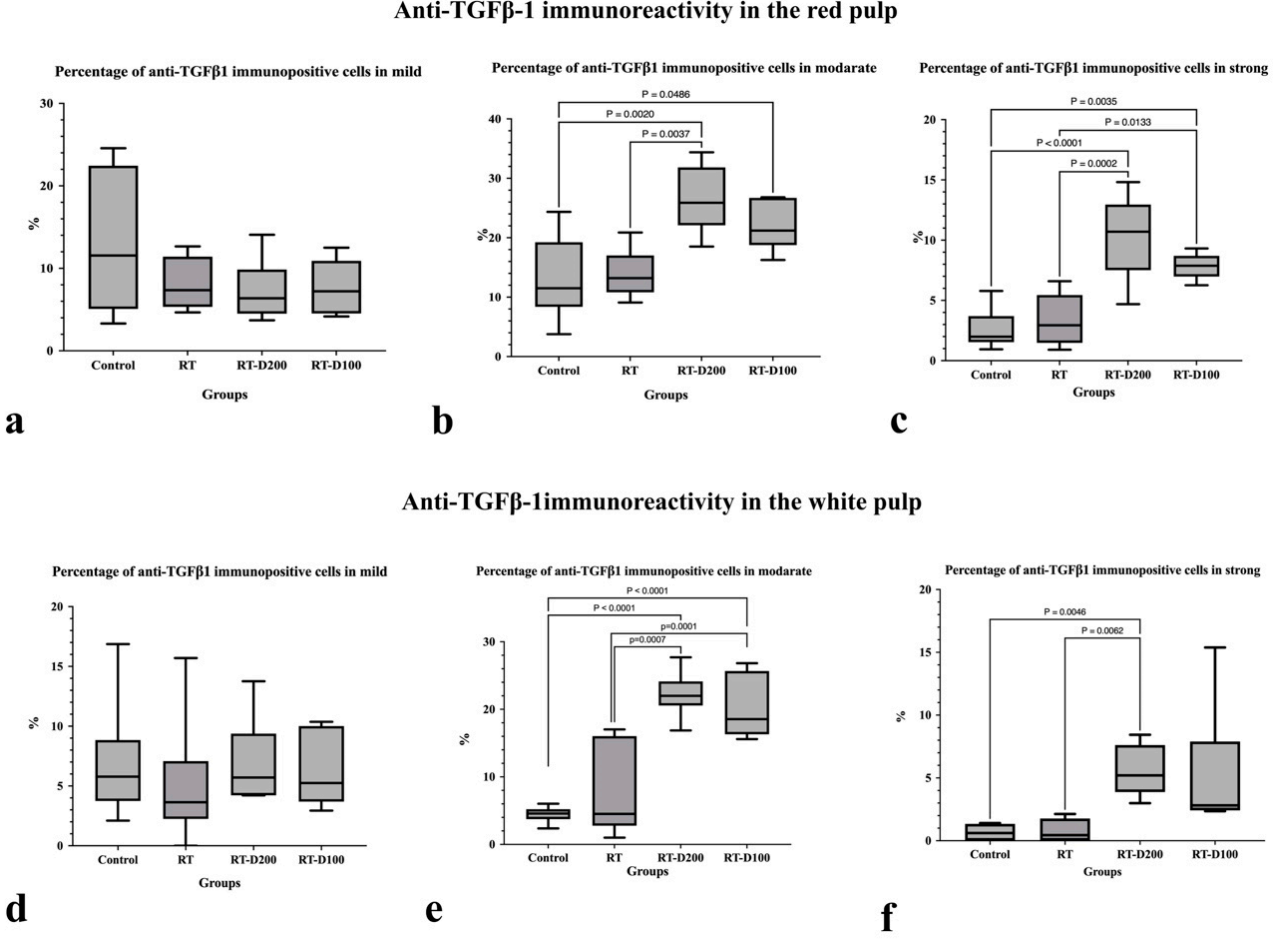
(**a**–**f**) Graphs containing anti-TGFß1 immunoreactivity assessment of all groups. (**b**,**c**,**e**,**f**) Differences between groups at the *p* < 0.05, *p* < 0.01, and *p* < 0.0001 levels are shown in the graph.

Additionally, no significant difference was observed between the groups in terms of the percentage of mildly anti-TGFβ1 immunopositive cells in the red pulp (Figure 5 and Figure 6a). Moderate positivity was significantly increased in the RT-D200 group compared to the RT group (*p* = 0.0037). Similarly, the percentage was significantly increased in the RT-D200 and RT-D100 groups compared to control group (respectively, *p* = 0.002; *p* = 0.0486) (Figure 5 and Figure 6b). Strong immunopositivity of lymphocytes and macrophages was significantly increased in the RT-D200 group compared to the RT and control groups (respectively, *p* = 0.0002; *p* < 0.0001), and the RT-D100 group also showed higher positivity compared to the RT and control groups (respectively, *p* = 0.0133; *p* = 0.0035) (Figure 5 and Figure 6c). The percentage of mildly immunopositive cells in the white pulp region did not differ significantly between the groups (Figure 5 and Figure 6d). Moderately TGFβ1 positivity increased significantly in the RT-D200 and RT-D100 groups compared to the RT group (respectively, *p* = 0.0007; *p* = 0.0001). Similarly, a significant increase in strong positivity was observed in the RT-D200 and RT-D100 groups compared to the control group (*p* < 0.0001) (Figure 5 and Figure 6e). Also, a significant increase in strong positivity was observed in the RT-D200 comparison with RT and control groups (*p* = 0.0062, *p* = 0.0046) (Figure 5 and Figure 6f). Anti-TGFβ1 staining was observed at both the cytoplasmic and nuclear levels in the Control group, while it was observed at the cytoplasmic level in the RT and RT-D100 groups. Conversely, anti-TGFβ1 staining was observed mostly at the nuclear level and in some areas at the cytoplasmic level in the RT-D200 group (Figure 5).

**Figure 7:**
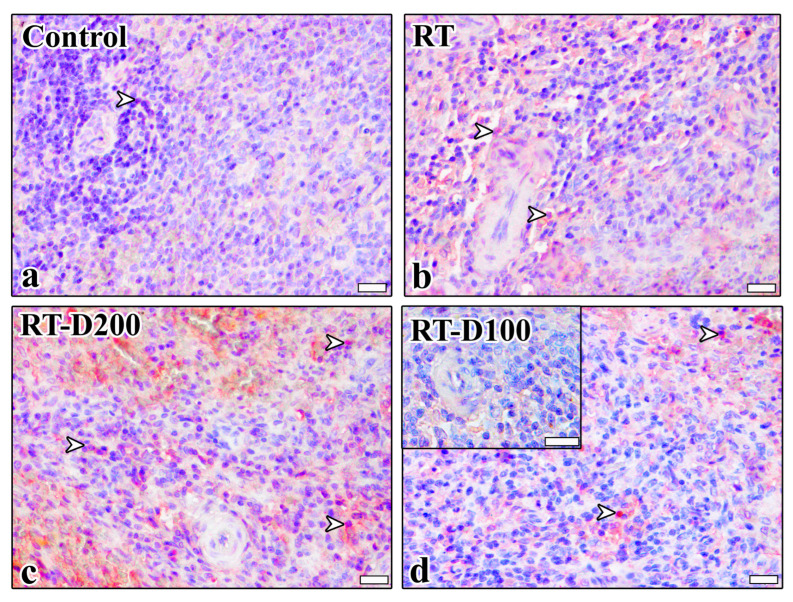
(**a**–**d**) Anti-TGF β3 immunoreactions are seen in all groups. Scale bars: 20 µm. Positive staining is indicated by arrowheads. Mayer’s hematoxylin was used for counterstaining. A semi-quantitative scoring system was used for evaluation: <10% = negative, 10–29% = mild, 30–59% = moderate, 60–100% = severe immunopositive cells.

**Figure 8:**
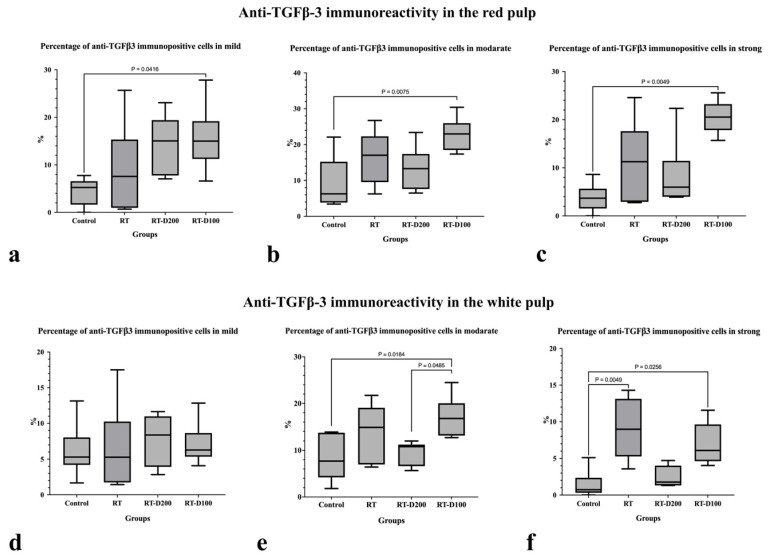
(**a**–**f**) Graphs containing anti-TGFß3 immunoreactivity assessment of all groups. (**a**–**c**,**e**,**f**) Differences between groups at the *p* < 0.05 and *p* < 0.01 levels are shown in the graph.

When anti-TGF-β3 immunoreactivity was examined, the percentage of TGF-β3-positive cells in the red pulp showed a significant increase in strong positivity in the RT-D100 group compared to control group (*p* = 0.0416) (Figure 7 and Figure 8a). Similarly, moderate immunoreactivity was significantly increased in the RT-D100 group compared to the control group (*p* = 0.0075) (Figure 7 and Figure 8b). Strong positivity was significantly increased in the RT-D100 group compared to the control group (*p* = 0.0049) (Figure 7 and Figure 8c). The percentage of TGF-β3-positive cells in the white pulp did not differ significantly between the groups for mild positivity (Figure 7 and Figure 8d). A significant increase in moderate positivity was observed in the RT-D100 group comparison with control group (*p* = 0.0184), a decrease in the RT-D200 group compared to the RT-D100 group (*p* = 0.0485) (Figure 7 and Figure 8e). A significant increase in strong immunopositivity was found in the RT group compared to the control group (*p* = 0.0049). Similarly, a significant increase in strong positivity was observed in the RT-D100 group compared to the control group (*p* = 0.0256). Similarly, a (Figure 7 and Figure 8f). Anti-TGFβ3 reaction was observed at the nuclear level in the Control and RT groups, while nuclear staining was intense in the RT-D100 groups. Cytoplasmic staining was weak in this group. On the other hand, anti-TGFβ3 staining was observed mostly at the nuclear level and in some areas at the cytoplasmic level in the RT-D200 group. Anti-TGFβ3 staining was also predominantly nuclear in the RT-D100 group (Figure 7).

### 3.3. Biochemical Analyses

**Figure 9:**
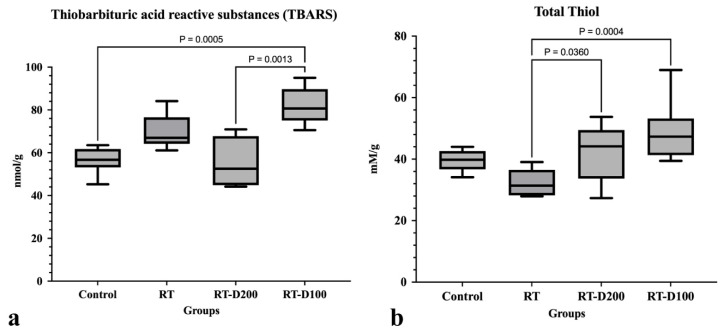
(**a**,**b**) Graphs containing biochemical reactivity assessment of all groups. Differences between groups at the *p* < 0.05 and *p* < 0.01 levels are shown in the graph.

When TBARS levels were evaluated, TBARS levels, which are considered an indicator of oxidative stress, were significantly increased in the RT-D100 group compared to the control group (*p* = 0.0005). TBARS levels decreased in the RT-D200 group compared to the RT-D100 group (*p* = 0.0013). When total thiol levels were evaluated, total thiol levels, which indicate antioxidant capacity, were significantly lower in the RT group compared to the Dexmedetomidine treatment groups (respectively, with RT-D200 at level *p* = 0.0004, with RT-D200 at level *p* = 0.036) (Figure 9a,b).

## 4. Discussion

Radiotherapy, an effective option in cancer treatment, has various side effects due to the penetration of ionizing radiation into the tissues [27,28]. The current study aimed to demonstrate the potential destructive effects of 8 Gy RT on splenic tissue using histopathological, immunohistochemical, and biochemical markers. Furthermore, the therapeutic and prophylactic efficacy of dexmedetomidine administered at 100 and 200 µg/kg was analyzed against these potential destructive effects. Histopathological findings revealed a significant decrease in white pulp diameter in the RT group. However, decreased cellular density and the presence of congestion were noted. These effects highlight the immunosuppressive effects of radiation. The observed increase in white pulp diameter and improved tissue architecture following dexmedetomidine administration, particularly in the D100 group, suggest anti-inflammatory and tissue-protective properties of this agent. Previously, dexmedetomidine has been reported to maintain antioxidant enzyme levels and reduce reactive oxygen metabolites. Therefore, it provides effective protection against oxidative damage caused by ionizing radiation [29]. Following the administration of 100 µg/kg dexmedetomidine, TBARS were found to increase compared to the control group and compared to 200 µg/kg. TBARS is a parameter that measures the peroxidation products of polyunsaturated fatty acids in cell membranes, and an increase in this parameter is an indicator of the presence of lipid peroxidation [30]. Tissue lipid peroxidation increases with radiation-induced oxidative stress [31]. Treatment at a dose of 200 µg/kg is thought to be more effective in reducing lipid peroxidation than the lower dose. Total thiol levels are a marker of antioxidant defense capacity. Low total thiol levels are considered an indicator of increased oxidative stress because thiol groups protect cells from oxidative damage by interacting with free radicals [24]. The increase in total thiol levels in the treatment groups can be considered as evidence that dexmedetomidine strengthens antioxidant defense at low and high doses.

Histopathological results revealed a decrease in white pulp diameter in the RT-D200 group. This reduction in white pulp diameter may be a marker of decreased immune response capacity [32,33]. Dexmedetomidine, an alpha-2 adrenergic receptor agonist, plays a critical role in the inflammatory immune system. This agent activates the alpha-2 receptor in the presynaptic membrane, thereby regulating norepinephrine release. Dexmedetomidine’s suppression of the inflammatory response has been observed to enhance immune function [21]. In our study, we observed that high doses of dexmedetomidine suppressed white pulp diameter. Previously, this agent has been suggested to suppress the proliferation of CD4+ and CD8+ T cells in animal models [34].

Apoptosis is an important cascade of processes involved in the homeostasis of organs and tissues; it also plays an active regulatory role in the maturation of T and B lymphocytes in peripheral lymphoid organs after antigen recognition [35]. Caspase-3 is one of the important effector factors that mediate cell death. In this context, caspase-3 activation can be considered a marker of apoptosis [36]. Considering the findings regarding anti-caspase-3 immunoreactivity, ionizing radiation was found to induce a strong apoptotic effect in both red and white pulp. While dexmedetomidine suppressed apoptotic effects in red pulp at both doses, the antiapoptotic effect at low doses in white pulp was insufficient. In this context, low vascularization in the white pulp may have caused low-dose dexmedetomidine to not have a sufficient effect. On the other hand, lymphocyte subpopulations have been shown to exhibit different sensitivities even to radiation doses lower than 3 Gy. Generally, B lymphocytes have been reported to be more sensitive than T lymphocytes. Similarly, differences in the radiation sensitivity of lymphocytes in organs and circulation are observed due to tumor infiltration [37]. Considering the lymphocyte populations in the white pulp and red pulp [38], it is likely that the cells in the white pulp respond more sensitively to radiation and undergo apoptotic processes. We can suggest that dexmedetomidine is insufficient at low doses but protects T lymphocytes at 200 µg/kg. However, further dose-dependent studies are needed on this topic.

When the findings of our study regarding anti-TGF-β1 were examined, it was determined that the expression of TGF-β1, a fibrogenic cytokine important against radiation-induced tissue damage, in the red and white pulp was increased in the treatment groups compared to the RT group. While some studies in the literature provide evidence that dexmedetomidine suppresses TGF-β1 [39], the findings in our study contradict this information. It is plausible that low-dose dexmedetomidine, due to its anti-inflammatory effect, suppresses T and B lymphocytes in the spleen and induces TGF-β1 to repair radiation-induced damage. When examining the histopathological effects of 100 µg/kg dexmedetomidine, it was found that it failed to suppress fibrosis induced by radiotherapy, but considering that it suppressed fibrosis at higher doses, it may be suggested that 200 µg/kg dexmedetomidine provides more effective repair in the spleen via TGF-β1. Molecular studies are needed to address possible mechanisms.

TGF-β3 is a multifunctional cytokine that participates in many physiological processes, such as cell cycle regulation, immune regulation, and fibrogenesis. It is known that ionizing radiation used in cancer treatment affects many cells’ signaling pathways. One of these pathways is related to TGF-β3 [12]. TGF-β3, a cytokine involved in wound healing, acts to reduce scar formation [40]. It is noteworthy that TGF-β3 expression increased in the low-dose dexmedetomidine group. It is plausible that dexmedetomidine at a dose of 100 mg/kg contributes to the healing process by modulating stress to the extent that it triggers tissue regeneration, while 100 mg/kg suppresses this effect. This study suggests that, although TGF-β1 and TGF-β3 are biological members of the same superfamily, they shape the response in tissue regeneration and degeneration processes in opposite directions, while dexmedetomidine elicits dose-dependent differential responses to these biological factors. However, the underlying mechanisms need to be evaluated. The fact that 8 Gy radiation did not affect the levels of TGF-β1 and 3 in the spleen tissue may be due to radiation inducing proinflammatory processes such as TNF-α. This may be due to the inhibition of TGF-β1 levels because of the cross-reaction between the resulting cytokines [41,42,43,44,45]. TGF-β can elicit both early and late responses in the spleen after radiation exposure. By triggering apoptosis in the early phase, it may contribute to signs of fibrosis in the late phase. The lack of TNF-α levels is a limitation of this study. Future studies will examine in detail the effects of radiotherapy and dexmedetomidine as a treatment option on biological factors involved in inflammation. In our study, radiation therapy resulted in an increase in the expression of TGF-β1 and TGF-β3 in the splenic red and white pulp. This increase is likely due to the critical role TGF-β plays in normal tissue damage and signs of fibrosis processes [13]. Dexmedetomidine treatment significantly suppressed this increase and prevented TGF-β-related processes. This finding may suggest that dexmedetomidine has a dual protective potential, both regulating the early immune response and limiting fibrotic remodeling in the late phase by suppressing ROS-mediated TGF-β activation. Therefore, both inflammatory and fibrotic processes in the spleen may be modulated by dexmedetomidine.

The immunomodulatory feature of dexmedetomidine, one of the important targets of modern drug therapy, is the result of being a member of the alpha 2 receptor family, which is a member of the adrenergic receptor family [22]. Furthermore, dexmedetomidine has been reported to modulate sympathetic nerve activity to the spleen [46]. In this context, the radioprotective efficacy of dexmedetomidine is closely related to splenic innervation as well as cellular mechanisms. Due to the radiosensitive property of lymphocytes, the effect of radiation on the spleen may be associated with lymphopenia. Damage to lymphocytes in the spleen can lead to cell death and thus white pulp atrophy [47]. Dexmedetomidine activates apoptosis mechanisms by decreasing IL-1β/IL-6/TNF-α and activating PI3K/Akt. In this context, dexmedetomidine exerts an immunomodulatory effect by reducing both neurogenic and cellular ionizing radiation damage [48]. Our study found that dexmedetomidine exhibited protective properties against apoptotic effects, particularly in the white pulp of the spleen. Our findings are consistent with the knowledge that dexmedetomidine activates PI3K/Akt, known mechanisms that limit apoptosis and oxidative stress. Therefore, our findings suggest that dexmedetomidine protects the irradiated spleen through neuroimmune and intracellular pathways.

The main limitation of our study is that we did not assess apoptosis in immune cells using TUNEL staining. Caspase-3, which indicates that the cell has entered the apoptotic pathway, could have been a complementary approach to the TUNEL method, which indicates the late phase of apoptosis. More molecular studies are needed regarding the effects of radiation on the immune system. Another limitation of our study is the absence of a dexmedetomidine-only group. Since the main purpose of the study was to investigate the potential protective effect of dexmedetomidine against radiation-induced immune system damage, including such a group, was beyond the scope of the current design. Furthermore, given the existence of several studies on the protective effects of dexmedetomidine, we aimed to minimize the number of animals used, in accordance with the 3R (Replacement, Reduction, Refinement) principles. Future molecular studies on the effects of dexmedetomidine alone at the cellular level are needed to further clarify its independent effects.

## 5. Conclusions

In conclusion, this study demonstrates that histological damage and oxidative stress induced by radiotherapy in spleen tissue can be alleviated in a dose-dependent manner with dexmedetomidine. High-dose dexmedetomidine (200 µg/kg) appears to be more effective in reducing radiation-induced apoptosis, signs of fibrosis, and oxidative stress. These findings suggest that dexmedetomidine may be a potential protective agent in preventing radiation-associated immune damage.

## Data Availability

The corresponding author is able to provide the datasets that were generated and/or analyzed during the present study upon reasonable request.
